# Stimulating Memory: Reviewing Interventions Using Repetitive Transcranial Magnetic Stimulation to Enhance or Restore Memory Abilities

**DOI:** 10.3390/brainsci11101283

**Published:** 2021-09-28

**Authors:** Connor J. Phipps, Daniel L. Murman, David E. Warren

**Affiliations:** Department of Neurological Sciences, University of Nebraska Medical Center, Omaha, NE 68198, USA; connor.phipps@unmc.edu (C.J.P.); dlmurman@unmc.edu (D.L.M.)

**Keywords:** TMS, rTMS, memory, hippocampus, brain networks, non-invasive brain stimulation, mild cognitive impairment, Alzheimer’s disease

## Abstract

Human memory systems are imperfect recording devices that are affected by age and disease, but recent findings suggest that the functionality of these systems may be modifiable through interventions using non-invasive brain stimulation such as repetitive transcranial magnetic stimulation (rTMS). The translational potential of these rTMS interventions is clear: memory problems are the most common cognitive complaint associated with healthy aging, while pathological conditions such as Alzheimer’s disease are often associated with severe deficits in memory. Therapies to improve memory or treat memory loss could enhance independence while reducing costs for public health systems. Despite this promise, several important factors limit the generalizability and translational potential of rTMS interventions for memory. Heterogeneity of protocol design, rTMS parameters, and outcome measures present significant challenges to interpretation and reproducibility. However, recent advances in cognitive neuroscience, including rTMS approaches and recent insights regarding functional brain networks, may offer methodological tools necessary to design new interventional studies with enhanced experimental rigor, improved reproducibility, and greater likelihood of successful translation to clinical settings. In this review, we first discuss the current state of the literature on memory modulation with rTMS, then offer a commentary on developments in cognitive neuroscience that are relevant to rTMS interventions, and finally close by offering several recommendations for the design of future investigations using rTMS to modulate human memory performance.

## 1. Introduction

Human memory systems are understood to be imperfect recording devices, and the performance of these systems is negatively impacted by age and disease. Memory loss is the most common cognitive complaint in older adults, while clinically significant memory deficits exaggerate age-related trends and are often attributable to neurodegenerative disease. The most common form of pathological memory decline is dementia due to Alzheimer’s disease (AD) [1]. Unfortunately, current pharmacological interventions for AD-related memory impairment, such as cholinesterase inhibitors, offer limited benefit for memory loss [2,3]; this is also true of other interventions for AD such as lifestyle changes [4,5,6]. The lack of effective treatments for memory loss, AD-related or not, leaves a significant need unmet: memory loss has negative consequences for independence, autonomy, and identity. Efficacious treatments for memory loss could preserve these faculties [7,8,9]. Fortunately, recent findings suggest that targeted non-invasive brain stimulation (NBS) may offer meaningful opportunities for treatment [10]. Specifically, transcranial magnetic stimulation (TMS), a form of NBS, has been reported to improve memory in healthy younger adults, healthy older adults, and individuals with AD [11,12,13,14]. TMS may therefore hold promise as a potential symptomatic treatment for memory loss. Still, a review of the current literature reveals substantial variability in methods, outcome measures, and populations. Consistent with the methodological variability, findings from interventions using repetitive TMS (rTMS) to enhance memory have been inconsistent. To address this, our review seeks to summarize the results of recent rTMS studies in patients on the AD continuum, discuss potential sources of heterogeneity, and provide suggestions on how the field could enhance rigor and reproducibility in future work. 

## 2. Review of Prior Work

### 2.1. Organization of the Review

We performed a narrative review based on searches of commonly used databases indexing scholarly works (PubMed, google scholar). Following the primary identification of literature, forward and backward citation searches were carried out for studies in this initially identified body. We observed that investigations testing TMS as a tool for memory enhancement or a treatment for memory loss have varied widely in their approaches. Acknowledging this heterogeneity, we identified two key independent variables that were used to organize our review: first *stimulation site* and then *target population*. As with the independent variables, we noted that outcome measures could similarly be divided into *changes in cognitive abilities (memory, executive functions, etc.)* and *changes in brain variables (structure and/or function)*. For a summary of rTMS studies organized and annotated according to these attributes, please refer to Table 1.

Regarding *stimulation sites*, investigators have most often selected rTMS targets within frontal or parietal association areas. Importantly, these regions are located immediately beneath the skull and thus within the limited range of typical TMS systems (~2–3 cm beneath the scalp) [15]. Within the brain’s frontal lobe, studies have frequently targeted dorsolateral prefrontal cortex (dlPFC). This popularity of dlPFC as a stimulation target may be attributable to its known contributions to many cognitive processes including working memory [16,17,18,19]. dlPFC is, of course, also a common rTMS target for clinical treatment of psychiatric disorders such as major depression [20]. More broadly, dlPFC is generally acknowledged as a brain region that is both feasible and safe for rTMS [21]. A less common alternative for rTMS has been the parietal lobe, and within it, rTMS studies have most frequently targeted posterolateral parietal cortex or angular gyrus (AG) [13,22]. In studies of rTMS and memory, AG has frequently been targeted because of its connectivity, both structural and functional, to medial temporal lobe regions which support declarative/relational memory processes. Further, AG is thought to be part of a large-scale intrinsic brain network, the default mode network (DMN) which has been implicated in normal memory function [23,24,25,26,27]. Additionally, the DMN is particularly impacted by AD [25,28,29], making modulation of DMN by rTMS a potentially intriguing therapy. For information on TMS mechanisms, refer to Box 1. 

Regarding *target populations*, while many studies of rTMS effects on memory have focused on healthy younger and healthy older individuals, there are an increasing number of studies investigating the potential for rTMS to treat memory loss within clinical populations (e.g. [30,31,32]). Studies using rTMS have also recruited individuals with clinical conditions that often precede AD, including (amnestic or non-amnestic) mild cognitive impairment (aMCI/MCI) [33,34,35].

In our review of published work, rTMS interventions for memory most frequently involved *frontal lobe* stimulation targets and *healthy individuals*, so we begin by summarizing findings from those studies.

### 2.2. rTMS of Frontal Lobe Sites

#### 2.2.1. rTMS of dlPFC: Healthy Young and Healthy Old

In the current literature, dlPFC has been stimulated with a variety of rTMS parameters and some reports suggest left and right dlPFC may respond differently to rTMS. Within studies targeting dlPFC in healthy adults considered in this review, the left hemisphere has been more frequently targeted. rTMS of left dlPFC has produced moderately consistent effects on resting-state functional connectivity (RSFC) but less consistent cognitive outcomes. Regarding cognitive changes associated with left dlPFC rTMS, eight of twelve studies reviewed here reported significant cognitive improvements associated with high-frequency stimulation [14,34,35,38,39,41,42,44,45,46,71,72]. However, and exemplifying the heterogeneity of outcomes in this domain, one study using low-frequency rTMS reported acute cognitive impairment [43]. 

Heterogeneity in rTMS methods and outcomes can be observed even in the limited domain of rTMS of left dlPFC of healthy adults. In one study, Chung and colleagues applied intermittent theta-burst stimulation (iTBS) rTMS targeting left dlPFC [41]. rTMS at 50%, 75%, and 100% motor threshold (MT) was associated with different results for each intensity. Their study observed a response similar to an inverted U-shaped curve, with no significant results at 50%, cognitive enhancement at 75%, and intermediate enhancement at 100%. In a similar study, Davis and colleagues applied 5 Hz rTMS at 120% MT to left dlPFC but observed no significant change in cognitive ability [42]. Together, these studies suggest that the greater the rTMS stimulation intensity does not strictly correspond to improved outcomes, and that there may be interactions of stimulation intensity and frequency on outcomes.

In two studies described above [41,42], rTMS was associated with changes in RSFC or EEG variables. Further, Davis and colleagues observed that RSFC changes were associated with better cognitive performance, including increased similarity in brain activation patterns during encoding and retrieval during a memory task [42,44]. 

Intriguingly, prior rTMS studies targeting dlPFC also suggest that brain state during rTMS may influence the brain’s response and related cognitive effects. That is, the same rTMS protocol may yield different effects when administered during task performance or at rest. In one study, Bakulin and colleagues applied rTMS to left dlPFC during different phases of a modified Sternberg task and observed differences in n-back performance were associated with phase of stimulation [39]. Specifically, the authors found that when rTMS was applied in absence of the modified task, 10 Hz rTMS to the left dlPFC was associated with significantly increased scores on the n-back task. Conversely, when rTMS was applied during any phase of the modified task, no significant benefit was observed. Other authors have speculated that rTMS during a task may invert the responses putatively associated with high-frequency and low-frequency rTMS [62,73,74]. While there is some evidence of efficacy differences between rTMS during task and rest, further study will be required to rigorously evaluate whether effects are truly inverted and if the same inversion is present for other stimulation targets.

Box 1 **Parameters and approaches for repetitive transcranial magnetic stimulation**

- TMS uses a powerful electromagnet to apply a focal, transient magnetic pulse to stimulate activity in the neurons of underlying gray matter [21]. When multiple TMS pulses are applied in series or in more complex temporal patterns, the procedure is called repetitive transcranial magnetic stimulation (rTMS). Initial research surrounding rTMS indicated transient effects associated with stimulation [21]. Critically, it has also been reported that rTMS can modify the brain’s intrinsic functional networks over extended periods [49,60,75].
- rTMS approaches can apply stimulation in simple series or in more complex patterns such as theta-burst stimulation (TBS). rTMS frequencies are typically described as either “excitatory” or “inhibitory” [21] as a function of stimulation frequency (1 Hz vs. 1 Hz, respectively). Classification into either form of rTMS is determined by changes observed in the motor evoked potential following rTMS to the primary motor cortex. “Excitatory” frequencies are reported to be associated with increases in brain activity while “inhibitory” frequencies are reported to be related to increased long-term depression of synaptic transmission. In TBS, pulse sequences are applied at frequencies and in patterns which putatively mirror neural oscillatory patterns associated with cognition [76,77]. The response to theta-burst rTMS varies by the rest period between stimulation. Despite the differences in rest periods between forms of TBS, the 50 Hz TBS is applied at a repeated 5 Hz frequency [78,79,80]. In continuous theta-burst stimulation (cTBS), pulses are applied during a 40 second period of stimulation followed by a short rest period. Alternatively, stimulation can be applied in 10 shorter periods consisting of a triplet of pulses followed by a rest period called intermittent theta-burst stimulation (iTBS). Application of TBS rTMS in different patterns can produce divergent effects on brain activity, cognition, and behavior [21]. iTBS has been hypothesized to be associated with promoting brain activity, while cTBS has been putatively associated with increased long-term depression of synaptic transmission [78,79,80].
- rTMS protocols can also apply different intensities of simulation often tailored to each individual subject. Stimulation intensity is often individualized by first gauging an individual’s *motor threshold* [21]. This involves measuring the elecotromyographic (EMG) response to single-pulse TMS of primary motor cortex in a distal muscle either at rest (resting motor threshold, RMT) or in flexion (active motor threshold, AMT) [21]. The TMS pulse causes the targeted corticospinal tract to fire and trigger an overt response in the target muscle. After the cortical area associated with the predetermined muscle of interest, frequently the abductor pollicis brevis of the right hand, is located, an adaptive stepwise procedure is used determine the individual’s RMT/AMT. This procedure is a guided titration of TMS intensities near the strength that caused the initial EMG response. For RMT, the target intensity is the minimum stimulation strength required to generate a 50 μV or greater peak-to-peak intensity in five of ten stimulations as measured by EMG. The active motor threshold is similar but employs a higher threshold, 200 μV. This higher threshold is required to determine the measured response is due to stimulation and not flexion-related noise in the EMG. Following the motor thresholding procedure, the intensity of the rTMS protocol can then be individualized so that, for example, all participants receive rTMS at 110% of their unique RMT.

#### 2.2.2. rTMS of dlPFC: MCI and AD

Although AD and MCI (especially aMCI) are often associated with clinical memory deficits, rTMS studies in these populations have frequently assessed general cognitive outcomes rather than memory-specific outcomes. Still, studies of rTMS in individuals with MCI and AD have yielded relatively consistent results. Much of this consistency may be derived from the greater homogeneity of rTMS parameters selected for studies of these populations versus studies of healthy individuals.

For example, in our survey of this literature, high-frequency rTMS was frequently used in studies of patients with MCI/AD. In several studies that applied high-frequency rTMS to left dlPFC, stimulation was associated with improved scores on one or more common cognitive assessments, including the MoCA, MMSE, and/or ADAS-Cog [14,34,36,45]. In a smaller number of studies, significant improvements were also reported on domain-specific assessments of memory abilities such as associative memory and relational memory [38,45,74,81]. Notably, all studies targeting dlPFC in clinically impaired individuals observed cognitive improvement in at least one domain. Specifically, improvements in AVLT, paired associate learning, MMSE, ADAS-cog, Rivermead Behavioral Memory Test, letter-number sequencing, association memory, recognition, logical memory, MoCA, and word/image association were observed following ten or more sessions of rTMS for cognitively impaired individuals [11,14,34,35,36,38,45]. 

In another study, Rutherford and colleagues recruited patients with AD and applied 20 Hz rTMS at 100% RMT to bilateral dlPFC (serially, one hemisphere at a time) across 13 sessions [11]. Of special note, longitudinal follow-up with participants revealed they had significantly attenuated rates of decline compared to participants randomly assigned to a control condition. Replication of this promising finding would be an important step toward generalization to clinical treatment. 

Finally, it has also been reported that low-frequency rTMS of right dlPFC was associated with cognitive improvement [33,82,83,84]. This finding may be interesting in the context of both healthy and pathological aging, as there is some evidence that right dlPFC exhibits hyperactivity associated with diminished cognitive performance [85,86].

#### 2.2.3. rTMS of Other Frontal Lobe Areas

While the dlPFC has been the most common target for rTMS in the frontal lobe, several other sites in frontal regions have also been targeted. Among these sites are precentral gyrus, middle frontal gyrus, and right medial frontopolar cortex. Jung and colleagues have explored the effects of 1 Hz rTMS to left and right precentral gyrus, two additional non-association areas [49]. They observed decreased connectivity between the DMN and the right motor network, the insular network, and the visual network attributable to rTMS. Additionally, rTMS during task engagement resulted in decreased connectivity between the DMN and the dorsal attention network and increased connectivity between the DMN and the frontoparietal network. 

Regarding right medial frontopolar cortex as a target, one study investigated the effects of single-session 20 Hz or 1 Hz rTMS [50]. In this instance, the authors reported RSFC changes associated with improved cognition for the 20 Hz stimulation group and changes associated with poorer cognition following 1 Hz rTMS in the low-frequency stimulation group. 

rTMS of middle frontal gyrus (MFG) has also been explored by Wang and colleagues [48]. This group administerd 10 Hz rTMS to either a left or right middle frontal gyrus target for two consecutive days. While improvements in hippocampal-dependent relational memory were found following stimulation of the right hemisphere target, no such changes were present following rTMS to the left site. Different effects of rTMS applied to left and right MFG could be attributable to laterality, but replication would be an important step to aid the interpretation of these findings.

Interestingly, a second study of rTMS applied to MFG used RSFC to determine an rTMS target. Lynch and colleagues applied a connectome-based approach to identify independent targets for each subject within right MFG based on within-network RSFC [47]. The authors applied a single session of cTBS rTMS to right MFG, and observed reduced working memory performance associated with stimulation. 

### 2.3. rTMS of Parietal Lobe Sites

#### 2.3.1. rTMS of AG: Healthy Young and Healthy Old

Outside of the frontal lobe, much of the association cortex accessible to typical TMS approaches lies in lateral portions of the parietal lobe. In the context of memory-related rTMS studies, locations in the inferior parietal lobule have been targeted most frequently. This is likely due to associations with memory task performance based on neuropsychological and neuroimaging studies [87,88,89,90]. In particular, left AG has been a popular choice for rTMS-based modulation of memory function.

rTMS of AG has proved fruitful for memory researchers, illustrated most clearly by the work of Voss and colleagues [12,22,54,61]. Angular gyrus is a cortical area within the effective range of rTMS that exhibits strong RSFC with hippocampus. By targeting a DMN component functionally connected to hippocampus, many researchers have applied rTMS measured the effects of rTMS on hippocampal-dependent memory function. In particular, Voss and colleagues have frequently demonstrated success in modulation of memory performance, brain activity, or both using a paradigm involving 20 Hz rTMS to left AG at 100% RMT [12,13,52,53,54,55,58,60]. The only significant source of heterogeneity within the studies using this paradigm was the number of rTMS sessions. 

The bulk of the 20 Hz rTMS studies from Voss and colleagues targeting left AG used five rTMS sessions [12,13,54,55,56,58]. Perhaps unsurprisingly, rTMS studies using five rTMS sessions with similar parameters frequently observed similar outcomes. These studies reported improvement in both measures of memory and RSFC. More specifically, RSFC changes associated with improved cognition are observed in the DMN. These consisted of strengthened RSFC between left AG and left hippocampus. In addition to these primary findings, it has also been reported that rTMS promotes hippocampal RSFC with DMN components beyond AG [12]. Consistent with a mechanistic explanation for rTMS effects on memory, these changes in RSFC were also accompanied by significant cognitive changes [12,13,54,55,56,58]. The aforementioned improvements in relational memory performance following rTMS were significantly greater compared to participants in the placebo-sham conditions. Similar increases in cognitive performance and changes in RSFC have also been observed under several rTMS protocols originating from the same group [55,91,92].

Dosage, operationalized as number of stimulation sessions, may be a key factor in determining the efficacy of AG rTMS as a memory-enhancing therapy. Several studies have varied rTMS dosing to investigate this relationship. In one study, Freedberg and colleagues observed that three, four, or five sessions of rTMS to left AG resulted in similar RSFC changes, but those authors did not assess changes in memory [60]. In a study with similar design but inconsistent findings, Hendrikse and colleagues reported finding no significant cognitive benefit following four rTMS sessions [52]. To explore a potential minimum threshold, a dose-finding study was carried out by Freedberg and colleagues. Those authors reported that a minimum of 5 rTMS sessions was required for reliable, statistically significant change in hippocampal-AG RSFC [51]. While these studies indicate that a minimum number of rTMS sessions may be necessary to obtain reliable effects, Hermiller and colleagues also reported a single session of cTBS rTMS was adequate to induce comparable changes in RSFC [53]. While 20 Hz rTMS studies suggest that three to five sessions may be required to generate reliable RSFC changes, the recent cTBS study reportedly requiring only a single session is intriguing. It seems possible that different stimulation frequencies or different sets of stimulation parameters may require a unique number of minimum rTMS sessions for significant changes to be observed. Future research exploring this possibility is warranted.

Right AG has also been targeted with rTMS. In a single study, Tambini and colleagues applied cTBS rTMS to right AG [61]. Following this, significant cognitive improvement was observed and coupled with RSFC changes. Unfortunately, this was the only study targeting right AG, and additional research into right AG rTMS in healthy individuals is warranted. 

#### 2.3.2. rTMS of AG: MCI and AD

Although results from healthy young and old adults demonstrate the potential for rTMS to improve memory abilities, similar findings have not been reported for AD and (a)MCI. While new clinical trials are proceeding at the time of this writing [93], only one recent study was identified applying rTMS to AG in individuals with mild to severe AD. Velioglu and colleagues administered ten sessions of 20 Hz rTMS at 100% MT to left AG [57]. Visual recognition memory performance and the clock drawing test improved after stimulation. Notably, the cognitive improvements were associated with changes in RSFC and, somewhat surprisingly, significant changes in other blood-derived, neurally-relevent biomarkers. Following rTMS, individuals were reported to have elevated blood brain derived neurotrophic factor measures and lower oxidative status measures. While intriguing, caution is warranted when interpreting these findings because biomarker measures derived from peripheral blood and CSF do not always exhibit strong correlation [94]. 

#### 2.3.3. rTMS of Other Parietal Lobe Sites

Several parietal regions beyond AG have been targeted with rTMS. The next most common stimulation site was precuneus [62,63,64]. As precuneus lies at the core of the default mode network [95], several studies have identified significant cognitive or brain changes following rTMS targeting precuneus.

One such study by Chen and colleagues applied ten sessions of 10 Hz rTMS to precuneus in individuals with subjective cognitive decline [63]. Following stimulation, these researchers observed significantly improved episodic memory and RSFC between precuneus and posterior hippocampus. Improvement in these domains is reminiscent of AG stimulation, mainly due to the notable hippocampal RSFC changes. A similar outcome was also reported by Koch and colleagues [64]. Here again, ten sessions of rTMS were administered but with 20 Hz stimulation. Following stimulation, the authors noted significant improvement in episodic memory coupled with changes in RSFC and EEG profiles. Several studies also targeted precuneus with low-frequency or cTBS rTMS and found transient impairments in memory or metacognition [62,96,97,98]. 

Two studies reported applying rTMS to superior parietal regions, and both reported cognitive changes in healthy young adults. Both studies reported outcomes consistent with expectations for high-frequency and low-frequency rTMS stimulation. Specifically, Wang and colleagues observed significant improvement in recalling face/word pairs following two sessions of 10 Hz rTMS of their target in superior parietal cortex [48]. Alternatively, Ribeiro and colleagues observed acute cognitive impairment following one session of 1 Hz of rTMS to superior parietal cortex [65]. Beyond association regions in the parietal cortex, post-central gyrus has also been targeted due to its functional connections with the insula [66]. Following five sessions of 10 Hz rTMS, Addicott and colleagues reported increased RSFC between the target and left insula. The directionality of these findings is consistent with the putative associations between high- and low-frequency stimulation and cognitive enhancement/impairment and in some instances required fewer than five stimulation sessions.

## 3. Multitarget Stimulation

While rTMS studies have most frequently targeted a single cortical region, some investigators have also tested the effect of multitarget rTMS. As the name suggests, multitarget rTMS involves targeting multiple, distal brain regions for stimulation within the same paradigm either serially or, less often, simultaneously. The potential benefits of multitarget stimulation include modulation of brain activity in locations in one or more functional brain networks, and this approach could provide additive or interactive cognitive enhancement [99].

For example, one study employing multitarget stimulation serially targeted several temporal and parietal stimulation locations [100]. Here, the researchers used 20 Hz stimulation over frontal and parietal targets every weekday for six weeks. Following stimulation, adults with AD exhibited a significant increase in ADAS-cog performance, and there was evidence that this effect endured for up to 12 weeks. The reported durability of this improvement is unusual in the literature and could reflect lasting modulation of underlying functional brain networks.

The “neuroAD protocol” is another line of research using a multitarget rTMS approach [68,69,101,102]. The protocol involves stimulation of six distinct targets regions: left and right dlPFC, left and right somatosensory association cortex, Broca’s area, and Wernicke’s area. Targeting these areas, the authors sought to improve multiple behaviorally relevant functional networks impacted by AD [70]. During each stimulation session, three of the six targets were serially stimulated.. Three different brain regions were selected for stimulation every session, with each site being stimulated in 15 sessions [101]. Stimulating at 100–110% RMT was associated with significant improvement in ADAS-Cog performance following rTMS [70]. Meanwhile, stimulation at 90% RMT reported observed increases in MMSE scores [103]. Unique among rTMS therapies for memory, the neuroAD protocol was recently submitted to the FDA for consideration as an intervention for patients with MCI or AD. At the time of writing, the most recent FDA determination was that the cognitive benefits were not substantial enough to warrant approval due to their modest efficacy (less than a three-point improvement on ADAS-Cog) [104]. Regarding concerns about the protocol’s efficacy, it is possible that the limited magnitude of cognitive benefit associated with the protocol could be due to the inclusion of individuals with substantial AD-related cognitive impairment. For individuals with more mild impairment, evidence of greater cognitive improvement was present: nearly a third of these individuals improved by four or more points on the ADAS-Cog [70]. If upheld, this finding would suggest that the neuroAD intervention is more effective in earlier disease stages, such as MCI, rather than AD. 

Where the neuroAD protocol targeted several locations serially during a session, the development of new TMS coils has also allowed stimulation of multiple cortical areas simultaneously. The ability to broadly stimulate bilateral frontal, temporal, and parietal areas has been explored with “H”-style TMS coils. Specifically, 10 Hz rTMS has been applied using an H coil for twelve consecutive sessions in individuals with AD [67]. Improvements were noted in ADAS-Cog scores but not in several other measures (MMSE, depression, or caregiver ratings of subjective improvement). 

## 4. Developments Relevant to Treating Memory loss with rTMS

Approaches using rTMS to treat memory loss have evolved substantially over the last two decades, as have insights from neuroscience regarding functional brain organization, neurodegenerative diseases, and brain mechanisms supporting memory processes. These developments are important considerations for investigators designing new rTMS interventions for memory loss. Furthermore, the integration of key concepts into new paradigms could improve the efficacy and reproducibility of future rTMS research. Here, we review some key developments including acknowledgment of the brain’s large-scale functional networks, computational modeling of rTMS stimulation fields, and frequency-specific effects of rTMS.

### 4.1. Functional Brain Networks

The last decade has seen a tremendous expansion of the field’s understanding of the brain’s intrinsic functional organization. Readily identifiable, large-scale functional brain networks have been reliably observed both in group studies and at the level of individual participants [105,106]. This development may offer benefits for rTMS approaches similar to those provided by stereotactic alignment of structural MRI data with the physical brain: improved rigor and reproducibility through precision alignment to previously identified stimulation targets. Here, a key concept is the identification of stimulation targets using individualized maps of functional networks overlaid onto the physical brain. Similar targeting has already been applied with success in rTMS studies seeking to treat depression [107,108]. If implemented, this approach could supplement and refine earlier approaches that identify targets based on physical distance, gross neuroanatomical landmarks, or coordinate-based targets derived from brain atlases. 

Acknowledging functional network architecture in the design of rTMS interventions will help to ensure that the same functional network is being stimulated across different participants. For example, while dlPFC has shown promise as an rTMS target for treating memory loss [35,41,74], dlPFC is a large region of association cortex which includes several distinct functional networks [105,106]. Furthermore, the territory of these networks varies between individuals [109,110]. Stimulation of the same dlPFC location based on neuroanatomy or template-derived coordinates could therefore affect a different selection of functional networks between subjects unless targets are selected for each participant according to their brain’s unique functional organization.

A related consideration is that stimulation of different functional networks would be expected to affect different cognitive processes. A strong implication of rTMS not guided by functional network consideration is that cognitive benefits of rTMS interventions could vary between individuals as a function of the stimulated networks rather than stimulation efficacy per se. Alternatively, otherwise similar cognitive benefits might be attributable to changes in different cognitive processes between individuals. Taking memory performance as an example, deficits in executive functions [111,112] or depressed mood [113] have been associated with memory impairments, so by inference, rTMS-associated improvements in executive functions or mood might be expected to enhance apparent memory performance, but without affecting underlying memory processes. While positive outcomes for patients are always welcome, interpretation of this type of finding could be confounded if superficially similar outcomes are attributable to different mechanisms. Integration of functional neuroimaging data into new TMS protocols to support network-specific targeting could help to avoid this specific confound.

While integration of functional neuroimaging data in rTMS intervention design is expected to enhance rigor, approaches to processing neuroimaging data can vary greatly and affect interpretation. It has been well documented that even when using the same dataset, different groups can generate significantly different findings [114]. This is not surprising because the number of possible analysis paths available to investigators is enormous; one recent report estimated that a typical fMRI dataset might afford nearly 7,000 unique analysis pipelines [115]. Thorough documentation of all steps of functional neuroimaging analysis is therefore essential, and widely-used workflows for analysis might be considered. For example, the Human Connectome Project [116] provides a standardized “minimal preprocessing pipeline” for structural and functional MRI data that appears to deliver reliable results [117]. This and similar pipelines can provide investigators with a predetermined workflow for MRI data processing, ensuring that all groups perform the same steps in the same order. Also, adoption of a common approach to analyzing neuroimaging data could reduce a significant source of heterogeneity for rTMS interventions that include neuroimaging outcomes. 

### 4.2. Modeling of TMS Field Locale/Stimulation Strength

Selection of TMS stimulation sites can be refined by anatomical and functional considerations as described above, and recent advances in computational modeling of electrical fields induced by non-invasive brain stimulation techniques (including TMS but also transcranial electrical stimulation) may support still further enhancement. Tools such as the SimNIBS toolkit [118] allow researchers to model the induced magnetic and electrical fields for an individual brain based on structural imaging data. The models then estimate the spatial extent of brain tissue affected by each TMS pulse. These estimates are important when considering the anatomical focality of the stimulation produced by a set of TMS parameters.

Model estimates of stimulation extent may also help investigators to understand which functional brain networks are most likely to be affected by TMS at a specific location. In combination with processed functional neuroimaging data, stimulation models can highlight functional networks that are most likely to be affected by TMS at a specific location. New studies could clearly benefit from this approach, and previous studies might benefit retroactively if the necessary data (structural MRI, resting-state fMRI, stimulation coordinates, and stimulation intensity) were collected. 

### 4.3. Stimulation Frequency and Patterning

Historically, rTMS frequencies have sometimes been dichotomized into either “excitatory” or “inhibitory” stimulation [21] as a function of stimulation frequency (1 Hz vs. 1 Hz, respectively). Classification as excitatory or inhibitory has been driven by changes observed in the motor evoked potential following rTMS to the primary motor cortex. Unfortunately, this simple scheme for classification may be overly reductionist, not addressing potentially important complexities while limiting exploration of new rTMS protocols. 

We respectfully suggest that the current “excitatory vs. inhibitory” dichotomy might benefit from a different characterization: high-frequency vs. low-frequency stimulation. Our suggestion for revised terminology arises from the neurophysiology of TMS. Crucially, it is not the case that “excitatory” stimulation causes an overt response at the rTMS target while “inhibitory” stimulation suppresses this response. Rather, irrespective of stimulation frequency, some neurons at the target location depolarize, making “inhibitory” a mischaracterization of the stimulatory effect from the standpoint of a cellular response. Findings from active rTMS, or rTMS performed during task performance, also weigh against the historical labeling of rTMS protocols. Active rTMS has been reported to invert the expected rTMS response [21,40,119,120]. That is, during active rTMS, typical “inhibitory” rTMS protocols have been associated with improvements in cognitive performance in some cases, whereas the same protocol at rest would be associated with reduced performance. “Excitatory” protocols similarly have been reported to swap responses in active conditions further supporting that such classification may be improper. Finally, evidence from studies applying physiological considerations in rTMS protocol determination also suggests that these classifications may be unfitting. One example of the importance of physiological considerations is “inhibitory” rTMS to the right dlPFC. In this instance, it has been observed that following rTMS, episodic memory performance is reported to significantly increase despite the “inhibitory” classification of stimulation [33,84]. It is important to note that right dlPFC does exhibit increased connectivity associated with reduced cognition [85,86]. In this way, although the “inhibitory” protocol improved cognition, it may have also acted to reduce the associated increase in connectivity. From a RSFC standpoint, “inhibitory” rTMS may be properly named in this instance, but the opposing cognitive outcomes add unnecessary confusion to the rTMS field. In this way, the classification of rTMS frequencies into “excitatory” or “inhibitory” addresses few specific instances and may inaccurately map onto neurophysiological (or other) outcomes.

As recent studies have enriched our understanding of how brain tissues and brain networks respond to rTMS frequencies and patterns, investigators now have a larger menu of frequencies from which to choose along with a better understanding of likely effects on underlying brain activity. For example, high-frequency rTMS protocols have been associated with increased within-network connectivity of a targeted functional network [42,50]. This may be an important consideration for efficacy because in other work, stronger within-network connectivity has been associated with better cognitive outcomes in neurological disease such as stroke [121]. Meanwhile, low-frequency rTMS has sometimes been associated with decreases in within-network connectivity accompanied by increases in between-network connectivity [42,84]. While this association may not be as robust as the association of high-frequency rTMS with stronger within-network connectivity, the potential for frequency-dependent effects on connectomic measures presents exciting possibilities for basic and clinical research.

Regarding the effects of different frequencies within the “high” or “low” categories, little is known. Very few published studies have measured whether different rTMS frequencies with the same expected activation valence (e.g., high-frequency, 10 Hz vs. 20 Hz) produce different effects. Instead, published work has more often contrasted high and low frequencies or the same stimulation frequency at one stimulation location versus another [42,44,50]. This gap in the literature may be important because the few publications on the topic suggest that varying stimulation frequency can affect cognitive outcomes. In one important demonstration, rTMS at 20 Hz and iTBS were associated with different cognitive outcomes following one session of rTMS targeting left AG [53]. Future research on rTMS methods may help to titrate stimulation frequencies and patterns that combine continued safety with greater efficacy. For the immediate future, new rTMS interventions may benefit by simply acknowledging the expected strengthening of within-network connectivity associated with typical high-frequency rTMS.

## 5. Suggestions for Studies Using rTMS to Treat Memory Loss

While rTMS shows promise as a potential intervention to enhance declarative/relational memory abilities or to treat memory loss (age-related or pathological), substantial between-study heterogeneity in design has made direct comparisons difficult. Here, we will close our review by discussing study design features and rTMS parameters that we expect will enhance the rigor, reproducibility, and efficacy of new investigations. These include, but are not limited to, selecting a functional network to target, finding suitable stimulation locations within that network, thoughts on TMS coil placement, selection of rTMS frequency to utilize, numbers of rTMS sessions, and the importance of longitudinal follow-ups.

### 5.1. Stimulation Site Selection

Any rTMS study must select one or more stimulation sites. Predictably, stimulation at different sites has been associated with different cognitive and behavioral outcomes. Acknowledging this, studies focused on memory enhancement or treatment of memory loss should select one (or more than one) site previously associated with memory abilities. Based on prior work and insights from the normative functional organization of the brain, we offer two broad insights and several more specific recommendations.

Perhaps our strongest recommendation is that investigators should consider selecting targets based on functional network locations in addition to structural features or coordinates. The parallel, interdigitated nature of the brain’s functional networks [122] makes reliably targeting a specific network through structural features impractical. Conversely, functional targeting is a relatively simple enhancement that can be readily implemented [107,108]. Regarding which networks to target, two may be especially important for normal memory function [24,123]: the default mode network, which is often described as including the medial temporal lobes and hippocampus, structures essential for normal memory; and the frontoparietal network [90], which has been frequently implicated in fMRI studies observing “subsequent memory effects” (increases in activation related to remembered versus forgotten items). Importantly, functionally determined rTMS targets could potentially be derived from resting-state or task-based neuroimaging data (or both); each offers advantages. Resting-state fMRI is relatively easy to collect from most populations and affords the opportunity to readily identify intrinsic networks [124,125,126]. Alternatively, task-based fMRI, perhaps collected during memory task performance, might offer even more refined targets because of the direct association with memory performance [127]. In either case, individualized stimulation targets derived from analysis of functional neuroimaging data are strongly predicted to provide more consistent results than other approaches.

Turning to specific cortical locations, one possibility is the left posterior lateral parietal lobule, or more specifically, left AG. Left AG is a region of association cortex that has well-characterized structural connections with the medial temporal lobe and RSFC with the hippocampus [22]. This connectivity and the necessity of hippocampus for normal memory functions [26,128] make left AG an appealing target. As reviewed here, significant prior work has demonstrated that rTMS of left AG can improve declarative/relational memory in healthy young and healthy older participants [12,13,51,58]. Additionally, stimulation of left AG does not have any known association with relief from depressive symptoms or executive functions, potential confounds related to stimulating other sites (e.g., dlPFC).

rTMS of left dlPFC has also been previously associated with improved memory performance. However, the above concerns regarding potential confounds related to mood and executive functions may apply to stimulation of this region. Irrespective of which location is selected, we strongly recommend individual targeting of a specific functional network rather than a location guided by simple distance, neuroanatomical features, or transformed atlas coordinates. 

### 5.2. Stimulation Site Targeting

Less complex but no less important than selection of a stimulation site is targeting of the stimulation site during an rTMS session. Earlier methods using EEG or scalp landmarks [37,65,100] can be substantially improved upon by TMS instruments that support real-time stereotactic alignment of structural MRI data and the participant’s physical brain [53,56,58,129]. Extending the same stereotactic coordinates to the TMS coil allows accurate, reproducible targeting of a specific brain region during one or more TMS sessions. Recently, stereotactic localization of a target brain region has been further enhanced by robotic systems that can maintain precise head-coil positioning to account for head motion during rTMS sessions [129]. Whether automated or manual, stereotactic alignment systems substantially enhance experimental rigor for rTMS studies..

### 5.3. Frequency Selection

rTMS frequencies and protocols are dichotomized into “excitatory” (high-frequency and iTBS) or “inhibitory” (low-frequency and cTBS) frequencies [21]. While this dichotomy captures some important differences, factors beyond rTMS frequency also contribute the excitatory or inhibitory influence of rTMS. One such factor is the underlying physiology of the rTMS target and the functional network to which it belongs. rTMS of right dlPFC is a prime example of the role target physiology can play. Multiple reports suggest that 1 Hz rTMS of right dlPFC caused significant improvement in cognitive abilities [33,82,83,84]. That might be consistent with an “excitatory” influence of an “inhibitory” frequency. Whatever the underlying mechanism, this outcome exemplifies the complex relationship between rTMS parameters and cognitive outcomes.

Neurophysiological considerations may also provide insight into what rTMS frequencies may generate potent responses. For example, Chung and colleagues investigated whether iTBS at a frequency matched to an individual’s brain activity would outperform the “excitatory” 50 Hz iTBS rTMS [130]. While both the individual and 50 Hz iTBS were reported to significantly improve cognition, individualized iTBS was also associated with significant changes in EEG measures. These reports illustrate the potential impact of neurophysiological considerations on rTMS outcomes. Stimulation frequency is an rTMS parameter that could benefit from more study, including refinement of methods for determining individualized stimulation frequencies based on observed neurodynamics of a given brain.

### 5.4. Number of Sessions

Perhaps the greatest degree of consensus in the rTMS literature lies in the number of rTMS sessions necessary for reliable memory enhancement. Specifically, multiple consecutive days of rTMS appear to be necessary to reliably observe improvements in memory performance that endure for one or more days after stimulation. Regarding the absolute number of sessions required, some research has been conducted with the explicit goal of dose estimation. Following up on prior work that tested the effects of rTMS applied to left AG, one studied estimated that a minimum of five sessions was required for benefits to memory performance [51], while a similar study by the same group estimated that as few as three simulation sessions was adequate to observe significant changes in RSFC between the stimulation site in left AG and the hippocampus [60]. To the best of our knowledge, these two studies are the only published works examining the effects of different numbers of rTMS sessions for left AG rTMS. More research on dosing of rTMS to treat memory impairment would be helpful. However, based on these dose-finding studies and other studies reporting significant changes after left AG stimulation, a minimum of five stimulation sessions appears to be a reasonable criterion [51,60]. Notably, ongoing clinical trials in patients with MCI or AD may incorporate even more sessions, such as the “20 weekday sessions during a period of 2 to 4 weeks” in a trial by Taylor and colleagues [93].

### 5.5. Longitudinal Follow-Up

rTMS therapies for memory would be most beneficial if the effects endured for some prolonged period after stimulation. Unfortunately, many rTMS publications do not report longitudinal measures. Without longitudinal follow-up, the durability and dose-response curves of rTMS therapies are impossible to determine, and this creates challenges for future efforts to translate rTMS research to clinical applications. Collection of longitudinal follow-up measures, perhaps one, three, and six months after completion of an rTMS protocol, would be a welcome addition to the design of future studies. 

### 5.6. Methodological Heterogeneity Versus Discovery Science

We have noted the heterogeneous methodologies of rTMS interventions for memory, and we have suggested that this creates challenges for interpretation and generalization. In that context, the suggestions we offer in this section of our review are intended to highlight opportunities for investigators to enhance their study designs based on recent advances and best practices. However, we do not wish to promote a rigidly proscriptive methodological homogeneity; the field of rTMS for memory (or other cognitive) enhancement is much too young to suggest that any single approach is optimal. Discovery science and exploratory research remain essential to progress in rTMS interventions for memory. So, while departures from typical rTMS protocols should be well-justified, as long as they are conducted with great scientific rigor, such efforts may well prove effective, informative, or both. Standard approaches for rTMS will only be enhanced by novel efforts, and we fully expect that a review of best practices written a decade from now would differ significantly from our current work largely due to new basic science findings.

## 6. Conclusions

The brain systems that support declarative/relational memory are imperfect recorders that are negatively impacted by age and disease. Potential treatments for memory loss (or interventions to enhance memory performance) would be beneficial, and published work describing rTMS interventions offer preliminary evidence that non-invasive brain stimulation may offers symptom-modifying therapies. Our review of the current literature highlights many published examples of rTMS interventions that successfully modulated memory, often through multi-day high-frequency stimulation of regions in frontal or parietal association cortex. Unfortunately, the current rTMS literature suffers from significant heterogeneity which creates challenges for interpretation and comparison. To address this, we have offered suggestions for the design of future rTMS investigations with the goal of enhancing rigor and reproducibility. Our intent is not proscriptive; rather, we hope to encourage best practices that will speed the transition of rTMS-based memory modulation from laboratories to memory clinics where new therapies are sorely needed. By reducing methodological heterogeneity, introducing neuroimaging measures, and incorporating longitudinal follow-up, forthcoming memory-related rTMS studies have the opportunity to prove the method’s validity, generalizability, and translational potential to treat clinical memory loss. 

## Figures and Tables

**Table 1:** Properties of included studies.

Authors	Target	Intensity	Frequency	Sessions	Session Spacing (Days to Complete)	Cognitive Changes ([+/N/−] for rMTS) [Score Change]	Functional Connectivity Changes ([+/N/-] Area1:Area2)	Target Population	N
Frontal Lobe rTMS									
Cui et al. [36]	(R) dlPFC	90	10	10	CD(WD)	[+]AVLT	[+]PCC:(R)Fusiform Gyrus, [+]PCC:(L)Anterior Cingulate Gyrus	aMCI	25
Schluter et al. [37]	(R) dlPFC	110	10	1	NA	NA	[+]Salience network connectivity	H	15
Bagattini et al. [38]	(L) dlPFC	100	20	20	CD(WD)	[+]Paired-associate learning	NA	AD	50
Bakulin et al. [39]	(L) dlPFC	100	10	1	NA	[+]n-back	NA	HY	12
Beynel et al. [40]	(L) dlPFC	100	5	4	11	[+]Memory Manipulation	NA	H	85
Chung et al. [41]	(L) dlPFC	50	iTBS	1	NA	[N]n-back	[N]EEG	H	16
		75			NA	[+]n-back	[N]EEG		
		100			NA	[N]n-back	[N]EEG		
Davis et al. [42]	(L) dlPFC	120	1	1	NA	[N]Source Memory	[−]Changes in success related activity	HO	15
			5		NA	[N]Source Memory	[+]Changes in success related activity		
Fitzsimmons et al. [43]	(L) dlPFC	110	1	1	NA	[−]Set-shifting	[−]Task-based betweenness centrality of dlPFC	H	16
Li et al. [14]	(L) dlPFC	100	20	30	CD(WD)	[+]MMSE[2.03], [+]ADAS-Cog[-2.89]	[+]Plasticity Response at M1	AD	37
Drumond Marra et al. [35]	(L) dlPFC	110	10	10	CD(WD)	[+]Rivermead Behavioral Memory Test, [−]Logical Memory II , [+]Letter-number sequencing, [−]Trails B	NA	MCI	34
Schluter et al. [37]	(L) dlPFC	110	10	1	NA	NA	[−]Salience network connectivity	H	15
W.-C. Wang et al. [44]	(L) dlPFC	120	1	1	NA	[N]Associative memory	[N]Encoding and retrieval similarity	HO	14
			5		NA	[N]Associative memory	[+]Encoding and retrieval similarity		
Wu et al. [45]	(L) dlPFC	70	iTBS	14	CD(D)	[+]Association memory, [+]Recognition, [+]Logical Memory Test, [+]AVLT	[−](L) dlPFC:(R)Precuneus	AD	13
Xue et al. [46]	(L) dlPFC	90	20	1	NA	NA	[+]low-frequency fluctuation in Rostral Anterior Cingulate Cortex, [+]Rostral Anterior Cingulate Cortex:(L)Temporal Cortex	HY	38
Yuan et al. [34]	(L) dlPFC	80	10	20	CD(WD)	[+]MoCA	[+]ALFF for (R)Inferior Frontal Gyrus, (R)Precuneus, (L)AG, (R)Supramarginal gyrus	aMCI	12
Rutherford et al. [11]	(B) dlPFC	100	20	10(+3)	CD(WD)	[+]MoCA, [+]Word/image Association	NA	AD	10
Lynch et al. [47]	(R) Middle Frontal Gyrus	80	cTBS	1	NA	[−]n-back	NA	HY	24
H. Wang et al. [48]	(R) Middle Frontal Gyrus 1	100	10	2	CD(D)	[+]Face/word Pairs	NA	HY	8
	(L) Middle Frontal Gyrus 2				CD(D)	[+]Face/word Pairs	NA		
Jung et al. [49]	(L/R) Precentral Gyrus	100	1	1	NA	NA	[−]DMN activity when at rest	H	36
Riedel et al. [50]	(R) Medial Frontopolar cortex	100	1	1	NA	NA	[−](R)Medial Frontopolar cortex:Amygdala	HY	55
			20		NA	NA	[+(]R)Medial Frontopolar cortex:Amygdala		
Parietal Lobe rTMS									
Freedberg et al. [51]	(L) AG	100	20	4	CD(D)	NA	[+](L)AG:(L)Hippocampal Network	HY	6
Hendrikse et al. [52]	(L) AG	100	20	4	CD(D)	[N]Associative Memory	[−]Connectivity within (L)Hippocampal Network,	H	36
Hermiller, et al. [53]	(L) AG	80	cTBS	1	NA	[+]Word Recognition Memory	[+]Hipp:PCC, [+]Hipp:Left medial frontal Gyrus, [+]Hipp:Right Medialfrontal Gyrus	H	24
		80	iTBS		NA	[N]Word Recognition Memory	N		
		100	20		NA	[N]Word Recognition Memory	N		
Hermiller, et al. [54]	(L) AG	100	20	5	CD(D)	[+]Paired-associate learning, [N]Long-term forgetting	NA	HY	16
Kim et al. [55]	(L) AG	100	20	5	CD(D)	[N]Item recognition, [+]Contextual recollection	[+]Posterior-medial network activity	HY	16
Nilakantan et al. [56]	(L) AG	100	20	5	CD(D)	[N]Recollection Success, [+]Recollection Precision	[−]Late-positive evoked potential amplitude, [−]Theta-alpha oscillatory power	HY	12
Nilakantan et al. [13]	(L) AG	100	20	5	CD(D)	[N]Recollection Success, [+]Recollection Precision	[+]Recollection signals throughout the hippocampal-cortical network	HO	15
J.X. Wang Voss [12]	(L) AG	100	20	5	CD(D)	[+]Paired-associate learning	[+]Hipp:Posteior Hipp-cortical network	HY	16 *
Velioglu et al. [57]	(L) AG	100	20	10	14	[+]Wechsler Memory Scale-Visual	[−]Activity in Occipito-fusiform Gyrus, [−]Fusiform Gyrus:Precuneus, [−]Lateral Occipital Cortex:Precuneus, [+]Fusiform Gyrus:Frontal Opercular Cortex, [+]Lateral Occipital Cortex: Frontal Opercular Cortex	AD	11
J.X. Wang et al. [58]	(L) AG	100	20	5	CD(D)	[+]Paired-associate learning	[+]Cortical-hipp network connectivity	HY	16 *
Wynn et al. [59]	(L) AG	90	1	1	NA	[+]Delayed Recall Confidence	NA	H	25
Freedberg et al. [60]	(L) AG	100	20	3	CD(D)	NA	[+]Hipp:Precuneus, [+]Hipp:Fusiform Area, [+]Hipp:Lateral Parietal Area, [+]Hipp:Superior Parietal Area	HY	8
Tambini et al. [61]	(R) AG	80	cTBS	1	NA	[+]Associative memory success and confidence	Response was dependent on AG and Hippocampus connectivity	HY	25
Bonnì et al. [62]	Precuneus	100	cTBS	1	NA	[−]Source Memory Errors	NA	HY	30
Chen et al. [63]	Precuneus	100	10	10	CD(WD)	[+]AVLT	[−](L)Parahippocampal gyrus:Hipp memory network, [−](L)Middle temporal gyrus:Hipp memory Network	SCD	38
Koch et al. [64]	Precuneus	100	20	10	CD(WD)	[+]AVLT Delayed Recall[0.8]	[+]Beta band oscillations	PAD	14
Riberio et al. [65]	Superior Parietal Cortex	80	1	1	NA	[−]Spatial Working Memory	NA	HY	20
H. Wang et al. [48]	Superior Parietal Cortex	100	10	2	2	[+]Face/word Pairs	NA	HY	8
Addicott et al. [66]	(R) Postcentral Gyrus	100	10	5	CD(D)	NA	[+](R)Postcentral gyrus:(L)Insula	H	28
Multisite rTMS									
Leocani et al. [67]	(B) Frontal, Parietal, Temporal	120	10	12(+4)	3 sessions a week for 4 weeks	[+]ADAS-Cog[−1.01]	NA	AD	16
Rabey et al. [68]	neuroAD	90–110	10	30(+24)	CD(WD)	[+]ADAS-Cog[3.76]	NA	AD	15
Nguyen et al. [69]	neuroAD	100	10	30	CD(WD)	[+]ADAS-Cog	NA	MCI, AD	10
Sabbagh et al. [70]	neuroAD	110	10	30	CD(WD)	[+]ADAS-Cog([−0.32]	NA	AD	59

Information from studies reviewed here including authors, TMS target, stimulation intensity, stimulation frequency, number of rTMS sessions, whether cognitive changes were present, whether functional connectivity changes were present, the target population, and the number of subjects. Sessions within parentheses indicated maintenance sessions following intervention. Abbreviations: AD, Alzheimer’s disease; ADAS-Cog, The Alzheimer’s Disease Assessment Scale-Cognitive Subscale; AG, angular gyrus; aMCI, amnestic mild cognitive impairment; AVLT, Rey Auditory Verbal Learning Test; B, bilateral; CD, rTMS sessions on consecutive days; cTBS, continuous theta-burst stimulation; D, rTMS sessions took place daily; dlPFC, dorsolateral prefrontal cortex; EEG, significant EEG changes present; H, healthy; HO, healthy old; Hip, Hippocampus; HY, healthy young; iTBS, intermittent theta-burst stimulation; L, left; MCI, mild cognitive impairment; MMSE, Mini-Mental State Examination; MoCA, Montreal Cognitive Assessment; N, no change; NA, not applicable PAD, prodromal AD; R, right; SCD, subjective cognitive decline; WD, rTMS sessions took place on week days only; *, same set of participants; +, change associated with better cognition or positive change in resting-state functional connectivity; −, change associated with poorer cognition or negative change in resting-state functional connectivity.

## References

[1] Alzheimer’s Association (2021). 2021 Alzheimer’s disease facts and figures. Alzheimers Dement..

[2] Russ T.C., Morling J.R. (2012). Cholinesterase inhibitors for mild cognitive impairment. Cochrane Database Syst. Rev..

[3] Cummings J.L., Tong G., Ballard C. (2019). Treatment Combinations for Alzheimer’s Disease: Current and Future Pharmacotherapy Options. J. Alzheimers Dis. JAD.

[4] Moll van Charante E.P., Richard E., Eurelings L.S., van Dalen J.-W., Ligthart S.A., van Bussel E.F., Hoevenaar-Blom M.P., Vermeulen M., van Gool W.A. (2016). Effectiveness of a 6-year multidomain vascular care intervention to prevent dementia (preDIVA): A cluster-randomised controlled trial. Lancet.

[5] Vellas B., Carrie I., Gillette-Guyonnet S., Touchon J., Dantoine T., Dartigues J.F., Cuffi M.N., Bordes S., Gasnier Y., Robert P. (2014). Mapt study: A multidomain approach for preventing Alzheimer’s disease: Design and baseline data. J. Prev. Alzheimers Dis..

[6] Hampel H., Vergallo A., Aguilar L.F., Benda N., Broich K., Cuello A.C., Cummings J., Dubois B., Federoff H.J., Fiandaca M. (2018). Precision pharmacology for Alzheimer’s disease. Pharmacol. Res..

[7] Albert M.S., DeKosky S.T., Dickson D., Dubois B., Feldman H.H., Fox N.C., Gamst A., Holtzman D.M., Jagust W.J., Petersen R.C. (2011). The diagnosis of mild cognitive impairment due to Alzheimer’s disease: Recommendations from the National Institute on Aging-Alzheimer’s Association workgroups on diagnostic guidelines for Alzheimer’s disease. Alzheimers Dement..

[8] McKhann G.M., Knopman D.S., Chertkow H., Hyman B.T., Jack C.R., Kawas C.H., Klunk W.E., Koroshetz W.J., Manly J.J., Mayeux R. (2011). The diagnosis of dementia due to Alzheimer’s disease: Recommendations from the National Institute on Aging-Alzheimer’s Association workgroups on diagnostic guidelines for Alzheimer’s disease. Alzheimers Dement..

[9] Mol M.E.M., van Boxtel M.P.J., Willems D., Jolles J. (2006). Do subjective memory complaints predict cognitive dysfunction over time? A six-year follow-up of the Maastricht Aging Study. Int. J. Geriatr. Psychiatry.

[10] Freitas C., Mondragón-Llorca H., Pascual-Leone A. (2011). Noninvasive brain stimulation in Alzheimer’s disease: Systematic review and perspectives for the future. Exp. Gerontol..

[11] Rutherford G., Lithgow B., Moussavi Z. (2015). Short and Long-term Effects of rTMS Treatment on Alzheimer’s Disease at Different Stages: A Pilot Study. J. Exp. Neurosci..

[12] Wang J.X., Voss J.L. (2015). Long-lasting enhancements of memory and hippocampal-cortical functional connectivity following multiple-day targeted noninvasive stimulation. Hippocampus.

[13] Nilakantan A.S., Mesulam M.-M., Weintraub S., Karp E.L., VanHaerents S., Voss J.L. (2019). Network-targeted stimulation engages neurobehavioral hallmarks of age-related memory decline. Neurology.

[14] Li X., Qi G., Yu C., Lian G., Zheng H., Wu S., Yuan T.-F., Zhou D. (2021). Cortical plasticity is correlated with cognitive improvement in Alzheimer’s disease patients after rTMS treatment. Brain Stimulat..

[15] Deng Z.D., Lisanby S.H., Peterchev A.V. (2012). Electric fi eld depth–focality tradeoff in transcranial magnetic stimulation: Simulation comparison of 50 coil designs. Brain Stimulat..

[16] Velanova K., Jacoby L.L., Wheeler M.E., McAvoy M.P., Petersen S.E., Buckner R.L. (2003). Functional-anatomic correlates of sustained and transient processing components engaged during controlled retrieval. J. Neurosci. Off. J. Soc. Neurosci..

[17] Blumenfeld R.S., Ranganath C. (2006). Dorsolateral Prefrontal Cortex Promotes Long-Term Memory Formation through Its Role in Working Memory Organization. J. Neurosci..

[18] Mars R.B., Grol M.J. (2007). Dorsolateral Prefrontal Cortex, Working Memory, and Prospective Coding for Action. J. Neurosci..

[19] Barbey A.K., Koenigs M., Grafman J. (2013). Dorsolateral Prefrontal Contributions to Human Working Memory. Cortex J. Devoted Study Nerv. Syst. Behav..

[20] Center for Devices and Radiological Health (2021). Repetitive Transcranial Magnetic Stimulation (rTMS) Systems—Class II Special Controls Guidance for Industry and FDA Staff.

[21] Rotenberg A., Horvath J.C., Pascual-Leone A. (2014). Transcranial Magnetic Stimulation.

[22] Thakral P.P., Madore K.P., Schacter D.L. (2017). A Role for the Left Angular Gyrus in Episodic Simulation and Memory. J. Neurosci..

[23] Amodio D.M., Frith C.D. (2006). Meeting of minds: The medial frontal cortex and social cognition. Nat. Rev. Neurosci..

[24] Buckner R.L., Carroll D.C. (2007). Self-projection and the brain. Trends Cogn. Sci..

[25] Greicius M.D., Srivastava G., Reiss A.L., Menon V. (2004). Default-mode network activity distinguishes Alzheimer’s disease from healthy aging: Evidence from functional MRI. Proc. Natl. Acad. Sci. USA.

[26] Milner B. (2005). The medial temporal-lobe amnesic syndrome. Psychiatr. Clin. N. Am..

[27] Spreng R.N., Mar R.A., Kim A.S.N. (2009). The Common Neural Basis of Autobiographical Memory, Prospection, Navigation, Theory of Mind, and the Default Mode: A Quantitative Meta-analysis. J. Cogn. Neurosci..

[28] Fjell A.M., McEvoy L., Holland D., Dale A.M., Walhovd K.B. (2014). What is normal in normal aging? Effects of Aging, Amyloid and Alzheimer’s Disease on the Cerebral Cortex and the Hippocampus. Prog. Neurobiol..

[29] Seeley W.W., Crawford R.K., Zhou J., Miller B.L., Greicius M.D. (2009). Neurodegenerative diseases target large-scale human brain networks. Neuron.

[30] Weiler M., Stieger K.C., Long J.M., Rapp P.R. (2020). Transcranial Magnetic Stimulation in Alzheimer’s Disease: Are We Ready?. eNeuro.

[31] Chou Y., Ton That V., Sundman M. (2020). A systematic review and meta-analysis of rTMS effects on cognitive enhancement in mild cognitive impairment and Alzheimer’s disease. Neurobiol. Aging.

[32] Heath A., Taylor J., McNerney M.W. (2018). rTMS for the treatment of Alzheimer’s disease: Where should we be stimulating?. Expert Rev. Neurother..

[33] Turriziani P. (2012). Enhancing memory performance with rTMS in healthy subjects and individuals with Mild Cognitive Impairment: The role of the right dorsolateral prefrontal cortex. Front. Hum. Neurosci..

[34] Yuan L.-Q., Zeng Q., Wang D., Wen X.-Y., Shi Y., Zhu F., Chen S.-J., Huang G.-Z. (2021). Neuroimaging mechanisms of high-frequency repetitive transcranial magnetic stimulation for treatment of amnestic mild cognitive impairment: A double-blind randomized sham-controlled trial. Neural Regen. Res..

[35] Drumond Marra H.L., Myczkowski M.L., Maia Memória C., Arnaut D., Leite Ribeiro P., Sardinha Mansur C.G., Lancelote Alberto R., Boura Bellini B., Alves Fernandes da Silva A., Tortella G. (2015). Transcranial Magnetic Stimulation to Address Mild Cognitive Impairment in the Elderly: A Randomized Controlled Study. Behav. Neurol..

[36] Cui H., Ren R., Lin G., Zou Y., Jiang L., Wei Z., Li C., Wang G. (2019). Repetitive Transcranial Magnetic Stimulation Induced Hypoconnectivity Within the Default Mode Network Yields Cognitive Improvements in Amnestic Mild Cognitive Impairment: A Randomized Controlled Study. J. Alzheimers Dis. JAD.

[37] Schluter R.S., Jansen J.M., van Holst R.J., van den Brink W., Goudriaan A.E. (2018). Differential Effects of Left and Right Prefrontal High-Frequency Repetitive Transcranial Magnetic Stimulation on Resting-State Functional Magnetic Resonance Imaging in Healthy Individuals. Brain Connect..

[38] Bagattini C., Zanni M., Barocco F., Caffarra P., Brignani D., Miniussi C., Defanti C.A. (2020). Enhancing cognitive training effects in Alzheimer’s disease: rTMS as an add-on treatment. Brain Stimulat..

[39] Bakulin I., Zabirova A., Lagoda D., Poydasheva A., Cherkasova A., Pavlov N., Kopnin P., Sinitsyn D., Kremneva E., Fedorov M. (2020). Combining HF rTMS over the Left DLPFC with Concurrent Cognitive Activity for the Offline Modulation of Working Memory in Healthy Volunteers: A Proof-of-Concept Study. Brain Sci..

[40] Beynel L., Davis S.W., Crowell C.A., Hilbig S.A., Lim W., Nguyen D., Palmer H., Brito A., Peterchev A.V., Luber B. (2019). Online repetitive transcranial magnetic stimulation during working memory in younger and older adults: A randomized within-subject comparison. PLoS ONE.

[41] Chung S.W., Rogasch N.C., Hoy K.E., Sullivan C.M., Cash R.F.H., Fitzgerald P.B. (2018). Impact of different intensities of intermittent theta burst stimulation on the cortical properties during TMS-EEG and working memory performance. Hum. Brain Mapp..

[42] Davis S.W., Luber B., Murphy D.L.K., Lisanby S.H., Cabeza R. (2017). Frequency-specific neuromodulation of local and distant connectivity in aging and episodic memory function. Hum. Brain Mapp..

[43] Fitzsimmons S.M.D.D., Douw L., van den Heuvel O.A., van der Werf Y.D., Vriend C. (2020). Resting-state and task-based centrality of dorsolateral prefrontal cortex predict resilience to 1 Hz repetitive transcranial magnetic stimulation. Hum. Brain Mapp..

[44] Wang W.-C., Wing E.A., Murphy D.L.K., Luber B.M., Lisanby S.H., Cabeza R., Davis S.W. (2018). Excitatory TMS Modulates Memory Representations. Cogn. Neurosci..

[45] Wu X., Ji G.-J., Geng Z., Zhou S., Yan Y., Wei L., Qiu B., Tian Y., Wang K. (2020). Strengthened theta-burst transcranial magnetic stimulation as an adjunctive treatment for Alzheimer’s disease: An open-label pilot study. Brain Stimul. Basic Transl. Clin. Res. Neuromodul..

[46] Xue S.-W., Guo Y., Peng W., Zhang J., Chang D., Zang Y.-F., Wang Z. (2017). Increased Low-Frequency Resting-State Brain Activity by High-Frequency Repetitive TMS on the Left Dorsolateral Prefrontal Cortex. Front. Psychol..

[47] Lynch C.J., Breeden A.L., Gordon E.M., Cherry J.B.C., Turkeltaub P.E., Vaidya C.J. (2019). Precision Inhibitory Stimulation of Individual-Specific Cortical Hubs Disrupts Information Processing in Humans. Cereb. Cortex.

[48] Wang H., Jin J., Cui D., Wang X., Li Y., Liu Z., Yin T. (2020). Cortico-Hippocampal Brain Connectivity-Guided Repetitive Transcranial Magnetic Stimulation Enhances Face-Cued Word-Based Associative Memory in the Short Term. Front. Hum. Neurosci..

[49] Jung J., Bungert A., Bowtell R., Jackson S.R. (2020). Modulating Brain Networks with Transcranial Magnetic Stimulation Over the Primary Motor Cortex: A Concurrent TMS/fMRI Study. Front. Hum. Neurosci..

[50] Riedel P., Heil M., Bender S., Dippel G., Korb F.M., Smolka M.N., Marxen M. (2019). Modulating functional connectivity between medial frontopolar cortex and amygdala by inhibitory and excitatory transcranial magnetic stimulation. Hum. Brain Mapp..

[51] Freedberg M., Reeves J.A., Toader A.C., Hermiller M.S., Kim E., Haubenberger D., Cheung Y.K., Voss J.L., Wassermann E.M. (2020). Optimizing Hippocampal-Cortical Network Modulation via Repetitive Transcranial Magnetic Stimulation: A Dose-Finding Study Using the Continual Reassessment Method. Neuromodul. J. Int. Neuromodul. Soc..

[52] Hendrikse J., Coxon J.P., Thompson S., Suo C., Fornito A., Yücel M., Rogasch N.C. (2020). Multi-day rTMS exerts site-specific effects on functional connectivity but does not influence associative memory performance. Cortex J. Devoted Study Nerv. Syst. Behav..

[53] Hermiller M.S., VanHaerents S., Raij T., Voss J.L. (2019). Frequency-specific noninvasive modulation of memory retrieval and its relationship with hippocampal network connectivity. Hippocampus.

[54] Hermiller M.S., Karp E., Nilakantan A.S., Voss J.L. (2019). Episodic memory improvements due to noninvasive stimulation targeting the cortical–hippocampal network: A replication and extension experiment. Brain Behav..

[55] Kim S., Nilakantan A.S., Hermiller M.S., Palumbo R.T., VanHaerents S., Voss J.L. (2018). Selective and coherent activity increases due to stimulation indicate functional distinctions between episodic memory networks. Sci. Adv..

[56] Nilakantan A.S., Bridge D.J., Gagnon E.P., VanHaerents S.A., Voss J.L. (2017). Stimulation of the Posterior Cortical-Hippocampal Network Enhances Precision of Memory Recollection. Curr. Biol. CB.

[57] Velioglu H.A., Hanoglu L., Bayraktaroglu Z., Toprak G., Guler E.M., Bektay M.Y., Mutlu-Burnaz O., Yulug B. (2021). Left lateral parietal rTMS improves cognition and modulates resting brain connectivity in patients with Alzheimer’s disease: Possible role of BDNF and oxidative stress. Neurobiol. Learn. Mem..

[58] Wang J.X., Rogers L.M., Gross E.Z., Ryals A.J., Dokucu M.E., Brandstatt K.L., Hermiller M.S., Voss J.L. (2014). Targeted Enhancement of Cortical-Hippocampal Brain Networks and Associative Memory. Science.

[59] Wynn S.C., Hendriks M.P.H., Daselaar S.M., Kessels R.P.C., Schutter D.J.L.G. (2018). The posterior parietal cortex and subjectively perceived confidence during memory retrieval. Learn. Mem..

[60] Freedberg M., Reeves J.A., Toader A.C., Hermiller M.S., Voss J.L., Wassermann E.M. (2019). Persistent Enhancement of Hippocampal Network Connectivity by Parietal rTMS Is Reproducible. eNeuro.

[61] Tambini A., Nee D.E., D’Esposito M. (2018). Hippocampal-targeted Theta-burst Stimulation Enhances Associative Memory Formation. J. Cogn. Neurosci..

[62] Bonnì S., Veniero D., Mastropasqua C., Ponzo V., Caltagirone C., Bozzali M., Koch G. (2015). TMS evidence for a selective role of the precuneus in source memory retrieval. Behav. Brain Res..

[63] Chen J., Ma N., Hu G., Nousayhah A., Xue C., Qi W., Xu W., Chen S., Rao J., Liu W. (2020). rTMS modulates precuneus-hippocampal subregion circuit in patients with subjective cognitive decline. Aging.

[64] Koch G., Bonnì S., Pellicciari M.C., Casula E.P., Mancini M., Esposito R., Ponzo V., Picazio S., Di Lorenzo F., Serra L. (2018). Transcranial magnetic stimulation of the precuneus enhances memory and neural activity in prodromal Alzheimer’s disease. NeuroImage.

[65] Ribeiro J.A., Marinho F.V.C., Rocha K., Magalhães F., Baptista A.F., Velasques B., Ribeiro P., Cagy M., Bastos V.H., Gupta D. (2018). Low-frequency rTMS in the superior parietal cortex affects the working memory in horizontal axis during the spatial task performance. Neurol. Sci. Off. J. Ital. Neurol. Soc. Ital. Soc. Clin. Neurophysiol..

[66] Addicott M.A., Luber B., Nguyen D., Palmer H., Lisanby S.H., Appelbaum L.G. (2019). Low- and High-Frequency Repetitive Transcranial Magnetic Stimulation Effects on Resting-State Functional Connectivity between the Postcentral Gyrus and the Insula. Brain Connect..

[67] Leocani L., Dalla Costa G., Coppi E., Santangelo R., Pisa M., Ferrari L., Bernasconi M.P., Falautano M., Zangen A., Magnani G. (2020). Repetitive Transcranial Magnetic Stimulation With H-Coil in Alzheimer’s Disease: A Double-Blind, Placebo-Controlled Pilot Study. Front. Neurol..

[68] Rabey J.M., Dobronevsky E., Aichenbaum S., Gonen O., Marton R.G., Khaigrekht M. (2013). Repetitive transcranial magnetic stimulation combined with cognitive training is a safe and effective modality for the treatment of Alzheimer’s disease: A randomized, double-blind study. J. Neural Transm..

[69] Nguyen J.-P., Suarez A., Kemoun G., Meignier M., Le Saout E., Damier P., Nizard J., Lefaucheur J.-P. (2017). Repetitive transcranial magnetic stimulation combined with cognitive training for the treatment of Alzheimer’s disease. Neurophysiol. Clin. Clin. Neurophysiol..

[70] Sabbagh M., Sadowsky C., Tousi B., Agronin M.E., Alva G., Armon C., Bernick C., Keegan A.P., Karantzoulis S., Baror E. (2020). Effects of a combined transcranial magnetic stimulation (TMS) and cognitive training intervention in patients with Alzheimer’s disease. Alzheimers Dement. J. Alzheimers Assoc..

[71] Beynel L., Davis S.W., Crowell C.A., Dannhauer M., Lim W., Palmer H., Hilbig S.A., Brito A., Hile C., Luber B. (2019). Site-specific effects of online rTMS during a working memory task in healthy older adults. bioRxiv.

[72] Singh A., Erwin-Grabner T., Sutcliffe G., Paulus W., Dechent P., Antal A., Goya-Maldonado R. (2020). Default mode network alterations after intermittent theta burst stimulation in healthy subjects. Transl. Psychiatry.

[73] Bashir S., Al-Hussain F., Hamza A., Shareefi G.F., Abualait T., Yoo W.-K. (2020). Role of Single Low Pulse Intensity of Transcranial Magnetic Stimulation over the Frontal Cortex for Cognitive Function. Front. Hum. Neurosci..

[74] Cotelli M., Manenti R., Cappa S.F., Zanetti O., Miniussi C. (2008). Transcranial magnetic stimulation improves naming in Alzheimer disease patients at different stages of cognitive decline. Eur. J. Neurol..

[75] Hawco C., Voineskos A.N., Steeves J.K.E., Dickie E.W., Viviano J.D., Downar J., Blumberger D.M., Daskalakis Z.J. (2018). Spread of activity following TMS is related to intrinsic resting connectivity to the salience network: A concurrent TMS-fMRI study. Cortex J. Devoted Study Nerv. Syst. Behav..

[76] Buzsáki G. (2002). Theta oscillations in the hippocampus. Neuron.

[77] Buzsáki G., Draguhn A. (2004). Neuronal oscillations in cortical networks. Science.

[78] Huang Y.-Z., Edwards M.J., Rounis E., Bhatia K.P., Rothwell J.C. (2005). Theta burst stimulation of the human motor cortex. Neuron.

[79] Oberman L., Eldaief M., Fecteau S., Ifert-Miller F., Tormos J.M., Pascual-Leone A. (2012). Abnormal modulation of corticospinal excitability in adults with Asperger’s syndrome. Eur. J. Neurosci..

[80] Stagg C.J., Wylezinska M., Matthews P.M., Johansen-Berg H., Jezzard P., Rothwell J.C., Bestmann S. (2009). Neurochemical Effects of Theta Burst Stimulation as Assessed by Magnetic Resonance Spectroscopy. J. Neurophysiol..

[81] Cotelli M., Manenti R., Cappa S.F., Geroldi C., Zanetti O., Rossini P.M., Miniussi C. (2006). Effect of Transcranial Magnetic Stimulation on Action Naming in Patients With Alzheimer Disease. Arch. Neurol..

[82] Rami L., Gironell A., Kulisevsky J., Garcia-Sánchez C., Berthier M., Estévez-González A. (2003). Effects of repetitive transcranial magnetic stimulation on memory subtypes: A controlled study. Neuropsychologia.

[83] Sandrini M., Censor N., Mishoe J., Cohen L.G. (2013). Causal Role of Prefrontal Cortex in Strengthening of Episodic Memories through Reconsolidation. Curr. Biol..

[84] Turriziani P., Smirni D., Mangano G.R., Zappalà G., Giustiniani A., Cipolotti L., Oliveri M. (2019). Low-Frequency Repetitive Transcranial Magnetic Stimulation of the Right Dorsolateral Prefrontal Cortex Enhances Recognition Memory in Alzheimer’s Disease. J. Alzheimers Dis. JAD.

[85] Kumar S., Zomorrodi R., Ghazala Z., Goodman M.S., Blumberger D.M., Cheam A., Fischer C., Daskalakis Z.J., Mulsant B.H., Pollock B.G. (2017). Extent of Dorsolateral Prefrontal Cortex Plasticity and Its Association With Working Memory in Patients With Alzheimer Disease. JAMA Psychiatry.

[86] Joseph S., Zomorrodi R., Ghazala Z., Knezevic D., Blumberger D.M., Daskalakis Z.J., Mulsant B.H., Pollock B.G., Rajji T.K., Kumar S. (2020). Dorsolateral prefrontal cortex excitability assessed using TMS-EEG and its relationship with neuropsychiatric symptoms in Alzheimer’s dementia. Alzheimers Dement..

[87] Berryhill M. (2012). Insights from neuropsychology: Pinpointing the role of the posterior parietal cortex in episodic and working memory. Front. Integr. Neurosci..

[88] Berryhill M.E., Chein J., Olson I.R. (2011). At the intersection of attention and memory: The mechanistic role of the posterior parietal lobe in working memory. Neuropsychologia.

[89] Gilmore A.W., Nelson S.M., McDermott K.B. (2015). A parietal memory network revealed by multiple MRI methods. Trends Cogn. Sci..

[90] Uncapher M.R., Wagner A.D. (2009). Posterior parietal cortex and episodic encoding: Insights from fMRI subsequent memory effects and dual-attention theory. Neurobiol. Learn. Mem..

[91] Hermiller M.S., Chen Y.F., Parrish T.B., Voss J.L. (2020). Evidence for Immediate Enhancement of Hippocampal Memory Encoding by Network-Targeted Theta-Burst Stimulation during Concurrent fMRI. J. Neurosci..

[92] Warren K.N., Hermiller M.S., Nilakantan A.S., Voss J.L. (2019). Stimulating the hippocampal posterior-medial network enhances task-dependent connectivity and memory. eLife.

[93] Taylor J.L., Hambro B.C., Strossman N.D., Bhatt P., Hernandez B., Ashford J.W., Cheng J.J., Iv M., Adamson M.M., Lazzeroni L.C. (2019). The effects of repetitive transcranial magnetic stimulation in older adults with mild cognitive impairment: A protocol for a randomized, controlled three-arm trial. BMC Neurol..

[94] Laske C., Stransky E., Leyhe T., Eschweiler G.W., Maetzler W., Wittorf A., Soekadar S., Richartz E., Koehler N., Bartels M. (2007). BDNF serum and CSF concentrations in Alzheimer’s disease, normal pressure hydrocephalus and healthy controls. J. Psychiatr. Res..

[95] Andrews-Hanna J.R., Reidler J.S., Sepulcre J., Poulin R., Buckner R.L. (2010). Functional-Anatomic Fractionation of the Brain’s Default Network. Neuron.

[96] Hebscher M., Meltzer J.A., Gilboa A. (2019). A causal role for the precuneus in network-wide theta and gamma oscillatory activity during complex memory retrieval. eLife.

[97] Hebscher M., Ibrahim C., Gilboa A. (2020). Precuneus stimulation alters the neural dynamics of autobiographical memory retrieval. NeuroImage.

[98] Ye Q., Zou F., Lau H., Hu Y., Kwok S.C. (2018). Causal Evidence for Mnemonic Metacognition in Human Precuneus. J. Neurosci..

[99] Gu S., Pasqualetti F., Cieslak M., Telesford Q.K., Yu A.B., Kahn A.E., Medaglia J.D., Vettel J.M., Miller M.B., Grafton S.T. (2015). Controllability of structural brain networks. Nat. Commun..

[100] Zhao J., Li Z., Cong Y., Zhang J., Tan M., Zhang H., Geng N., Li M., Yu W., Shan P. (2017). Repetitive transcranial magnetic stimulation improves cognitive function of Alzheimer’s disease patients. Oncotarget.

[101] Andrade S.M., de Oliveira E.A., Alves N.T., Dos Santos A.C.G., de Mendonça C.T.P.L., Sampaio D.D.A., da Silva E.E.Q.C., da Fonsêca É.K.G., de Almeida Rodrigues E.T., de Lima G.N.S. (2018). Neurostimulation Combined With Cognitive Intervention in Alzheimer’s Disease (NeuroAD): Study Protocol of Double-Blind, Randomized, Factorial Clinical Trial. Front. Aging Neurosci..

[102] Rabey J.M., Dobronevsky E. (2016). Repetitive transcranial magnetic stimulation (rTMS) combined with cognitive training is a safe and effective modality for the treatment of Alzheimer’s disease: Clinical experience. J. Neural Transm..

[103] Gandelman-Marton R., Aichenbaum S., Dobronevsky E., Khaigrekht M., Rabey J.M. (2017). Quantitative EEG after Brain Stimulation and Cognitive Training in Alzheimer Disease. J. Clin. Neurophysiol. Off. Publ. Am. Electroencephalogr. Soc..

[104] Gaithersburg H. FDA Executive Summary Prepared for the March 21, 2019 Meeting of the Neurological Devices Panel 2019. https://www.fda.gov/advisory-committees/advisory-committee-calendar/june-3-4-2021-neurological-devices-panel-medical-devices-advisory-committee-meeting-announcement.

[105] Power J.D., Cohen A.L., Nelson S.M., Wig G.S., Barnes K.A., Church J.A., Vogel A.C., Laumann T.O., Miezin F.M., Schlaggar B.L. (2011). Functional network organization of the human brain. Neuron.

[106] Yeo B.T.T., Krienen F.M., Sepulcre J., Sabuncu M.R., Lashkari D., Hollinshead M., Roffman J.L., Smoller J.W., Zöllei L., Polimeni J.R. (2011). The organization of the human cerebral cortex estimated by intrinsic functional connectivity. J. Neurophysiol..

[107] Fox M.D., Buckner R.L., White M.P., Greicius M.D., Pascual-Leone A. (2012). Efficacy of transcranial magnetic stimulation targets for depression is related to intrinsic functional connectivity with the subgenual cingulate. Biol. Psychiatry.

[108] Fox M.D., Liu H., Pascual-Leone A. (2013). Identification of reproducible individualized targets for treatment of depression with TMS based on intrinsic connectivity. NeuroImage.

[109] Gordon E.M., Laumann T.O., Adeyemo B., Gilmore A.W., Nelson S.M., Dosenbach N.U.F., Petersen S.E. (2017). Individual-specific features of brain systems identified with resting state functional correlations. NeuroImage.

[110] Gordon E.M., Laumann T.O., Adeyemo B., Petersen S.E. (2017). Individual Variability of the System-Level Organization of the Human Brain. Cereb. Cortex.

[111] Malhotra P.A. (2019). Impairments of attention in Alzheimer’s disease. Curr. Opin. Psychol..

[112] Rizzo M., Anderson S.W., Dawson J., Myers R., Ball K. (2000). Visual attention impairments in Alzheimer’s disease. Neurology.

[113] Strömgren L.S. (1977). The influence of depression on memory. Acta Psychiatr. Scand..

[114] Botvinik-Nezer R., Holzmeister F., Camerer C.F., Dreber A., Huber J., Johannesson M., Kirchler M., Iwanir R., Mumford J.A., Adcock R.A. (2020). Variability in the analysis of a single neuroimaging dataset by many teams. Nature.

[115] Carp J. (2012). On the Plurality of (Methodological) Worlds: Estimating the Analytic Flexibility of fMRI Experiments. Front. Neurosci..

[116] Van Essen D.C., Ugurbil K., Auerbach E., Barch D., Behrens T.E.J., Bucholz R., Chang A., Chen L., Corbetta M., Curtiss S.W. (2012). The Human Connectome Project: A data acquisition perspective. NeuroImage.

[117] Glasser M.F., Sotiropoulos S.N., Wilson J.A., Coalson T.S., Fischl B., Andersson J.L., Xu J., Jbabdi S., Webster M., Polimeni J.R. (2013). The minimal preprocessing pipelines for the Human Connectome Project. NeuroImage.

[118] Thielscher A., Antunes A., Saturnino G.B. Field modeling for transcranial magnetic stimulation: A useful tool to understand the physiological effects of TMS?. Proceedings of the 2015 37th Annual International Conference of the IEEE Engineering in Medicine and Biology Society (EMBC).

[119] Cohen L.G., Celnik P., Pascual-Leone A., Corwell B., Falz L., Dambrosia J., Honda M., Sadato N., Gerloff C., Catalá M.D. (1997). Functional relevance of cross-modal plasticity in blind humans. Nature.

[120] Cohen L.G., Weeks R.A., Sadato N., Celnik P., Ishii K., Hallett M. (1999). Period of susceptibility for cross-modal plasticity in the blind. Ann. Neurol..

[121] Siegel J.S., Seitzman B.A., Ramsey L.E., Ortega M., Gordon E.M., Dosenbach N.U.F., Petersen S.E., Shulman G.L., Corbetta M. (2018). Re-emergence of modular brain networks in stroke recovery. Cortex J. Devoted Study Nerv. Syst. Behav..

[122] Braga R.M., Buckner R.L. (2017). Parallel Interdigitated Distributed Networks within the Individual Estimated by Intrinsic Functional Connectivity. Neuron.

[123] Buckner R.L., Andrews-Hanna J.R., Schacter D.L. (2008). The brain’s default network: Anatomy, function, and relevance to disease. Ann. N. Y. Acad. Sci..

[124] Lindquist M.A., Geuter S., Wager T.D., Caffo B.S. (2019). Modular preprocessing pipelines can reintroduce artifacts into fMRI data. Hum. Brain Mapp..

[125] Smith S.M., Beckmann C., Andersson J., Auerbach E.J., Bijsterbosch J., Douaud G., Duff E., Feinberg D.A., Griffanti L., Harms M.P. (2013). Resting state fMRI in the Human Connectome Project. NeuroImage.

[126] Wig G.S., Schlaggar B.L., Petersen S.E. (2011). Concepts and principles in the analysis of brain networks. Ann. N. Y. Acad. Sci..

[127] Davis S.W., Kragel J.E., Madden D.J., Cabeza R. (2012). The Architecture of Cross-Hemispheric Communication in the Aging Brain: Linking Behavior to Functional and Structural Connectivity. Cereb. Cortex.

[128] Cohen N.J., Squire L.R. (1980). Preserved learning and retention of pattern-analyzing skill in amnesia: Dissociation of knowing how and knowing that. Science.

[129] Neggers S.F.W., Langerak T.R., Schutter D.J.L.G., Mandl R.C.W., Ramsey N.F., Lemmens P.J.J., Postma A. (2004). A stereotactic method for image-guided transcranial magnetic stimulation validated with fMRI and motor-evoked potentials. NeuroImage.

[130] Chung S.W., Sullivan C.M., Rogasch N.C., Hoy K.E., Bailey N.W., Cash R.F.H., Fitzgerald P.B. (2019). The effects of individualised intermittent theta burst stimulation in the prefrontal cortex: A TMS-EEG study. Hum. Brain Mapp..

